# Mycetoma (Madura Foot): A Case Report of Misdiagnosis and Recovery in a Young Patient

**DOI:** 10.7759/cureus.94400

**Published:** 2025-10-12

**Authors:** Zomoroda Mohamed, Mohamedzain S Elamin, Mohammad Khalid, Salih Ahmed, Ahmed Galal Abdelmaged Mohamedahmed

**Affiliations:** 1 Surgical Anatomy, University of Bahri, Khartoum, SDN; 2 General Surgery, Royal College of Surgeons, Dublin, GBR; 3 Anatomy, University of Bahri, Khartoum, SDN; 4 Orthopedics and Trauma, University of Bahri, Khartoum, SDN; 5 General Surgery, Dongola Teaching Hospital, Dongola, SDN; 6 General Surgery, Al-Kamleen Teaching Hospital, Khartoum, SDN; 7 General Surgery, Royal College of Surgeons, Edinburgh, GBR

**Keywords:** diagnostic, madura foot, mycetoma foot, treatment, x-ray

## Abstract

Mycetoma is a chronic infection with a higher incidence in tropical and subtropical nations. We are presenting a rare case of mycetoma in a resident of Sudan, which is a temperate region, along with a short discussion. This case presentation discusses a 13-year-old male with a chronic foot discharge misdiagnosed as an abscess for two years. The condition, characterized by the development of a painless nodule leading to an open wound with purulent discharge, exemplifies the typical progression of actinomycetoma or eumycetoma. Following deep surgical debridement and antifungal therapy with itraconazole, the patient achieved complete recovery without recurrence. Mycetoma is a chronic granulomatous inflammatory process, typically with sinus tract formation secondary to fungal or bacterial pathogens. The most common is the forefoot infection. It is a slowly progressing disease of the deep dermis and subcutaneous tissue that can spread to the underlying bones. This case highlights the need for proper diagnosis and prompt aggressive management in the treatment of mycetoma, especially in endemic areas. Mycetoma needs to be diagnosed early and adequately, as well as aggressively managed to prevent functional as well as aesthetic damage.

## Introduction

Mycetoma is a chronic, specific, granulomatous, progressive, destructive inflammatory disease that involves the skin, subcutaneous tissues, and deeper structures. The causative organism may be true fungi, when the condition is called eumycetoma; when caused by bacteria, it is called actinomycetoma. The pathognomonic feature is the triad of painless subcutaneous mass, multiple sinuses, and seropurulent discharge. It causes tissue destruction, deformity, disability, and death in extreme cases [1].

The condition predominantly occurs in the “mycetoma belt” that lies between latitudes 15° south and 30° north, comprising the countries of Sudan, Somalia, Senegal, India, Yemen, Mexico, Venezuela, Colombia, Argentina, and a few others. The route of infection is inoculation of the organism resident in the soil through a traumatized area. Although in the vast majority there is no history of trauma, the portal of entry is always an area of minor, unrecognized trauma in a barefooted individual walking in a thorn-filled terrain. Hence, the foot is the most common site affected. Mycetoma is not contagious [1].

Mycetoma is a chronic infection. It is, however, not contagious. Following the onset of granuloma, the lesion expands further, and the overlying skin becomes stretched, smooth, glistening, and adherent. Foci of hyperpigmentation or hypopigmentation sometimes develop. Eventually, it penetrates deep tissue [1]. This is overall slow and late in eumycetoma. In actinomycosis, since mycetoma does not hurt, late presentations are common in the vast majority of cases. It is considered a slowly growing, painless, subcutaneous angioedema that most often occurs at the site of alleged trauma. The swelling is inconsistent in its physical appearance: hard and round, soft and lobulated, only occasionally cystic, and typically movable. Several secondary nodules may occur; these can become suppurated and drain through multiple sinus tracts. The sinuses may close briefly at discharge during the active phase of the disease. Fresh adjacent sinuses may open while some of the old ones may heal completely [1]. They cluster together and develop into abscesses, with discharge that may be serous, serosanguineous, or purulent. In the active phase of illness, sinuses discharge grains that may be red, yellow, white, or even black in color, depending on the organism.

Pain occurs when there is secondary bacterial infection [1]. The common sites affected are those that come into contact with soil during daily activities: the foot in 70% and the hand in 12%. In endemic regions, the knee, arm, leg, head, neck, thigh, and perineum are also affected. Mycetoma of the foot is the most common. The chest, abdominal wall, facial bones, mandible, testis, paranasal sinuses, and eye are uncommon sites [1].

In some patients, there may be areas of local hyperhidrosis over the lesion. This may be due to sympathetic overactivity or increased local temperature from raised arterial blood flow caused by chronic inflammation. In most patients, the regional lymph nodes are small and shotty. Lymphadenopathy is common and may result from secondary bacterial infection, lymphatic spread of mycetoma, or a local immune response to the disease [1]. The condition remains localized; constitutional disturbances are a sign of secondary bacterial infection. Cachexia and anemia from malnutrition and sepsis may be seen in late cases. It can be fatal, especially in cranial mycetoma [1].

Actinomycetes or fungi can cause mycetoma, a chronic subcutaneous infection characterized by granulomatous inflammation in the deep dermis and subcutaneous tissue, which may extend to the underlying bone [2]. The development of grains with aggregates of the causative organisms, which may be released onto the skin’s surface through several sinuses, is the defining feature of mycetoma [2]. In tropical and subtropical areas, where it can become endemic, this condition is common [2]. The feet and lower legs are among the body areas most frequently affected by mycetoma, and the infection usually appears on the dorsal portion of the forefoot [2].

## Case presentation

The patient, a 13-year-old male with no past medical history of diabetes, hypertension, or other chronic disease of medical importance, had an unhealed wound in the left foot. The condition began four years ago with the sudden appearance of a small foot nodule at the plantar aspect of the left foot, which developed into an open wound that became infected, exuding a white discharge with black granules. The systemic review was unremarkable.

**Figure 1:**
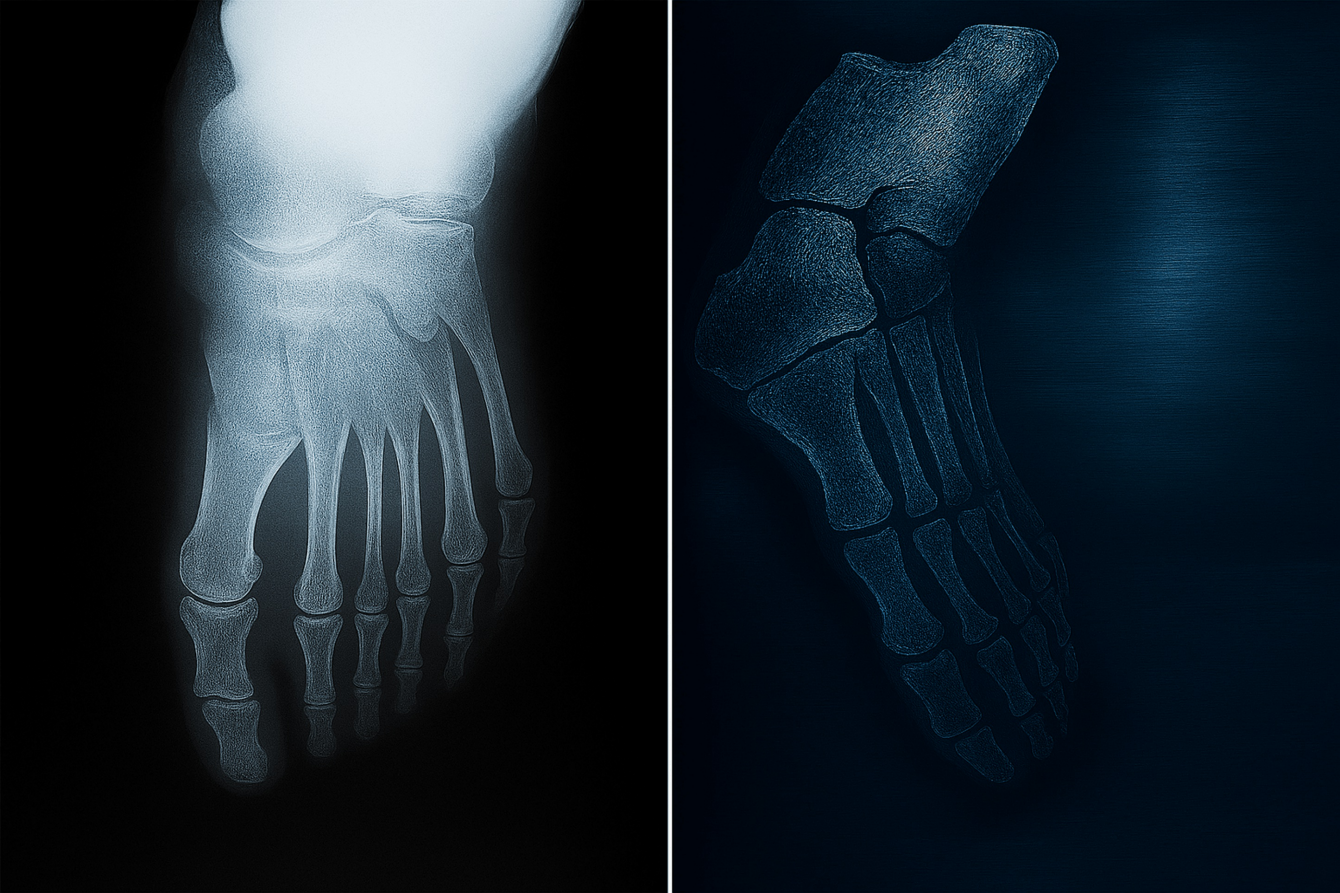
X-ray showing no abnormalities in the patient’s foot

The patient was initially misdiagnosed with a foot abscess, which was managed through two drainage procedures. Physical examination revealed persistent swelling with erythema at the plantar aspect of the foot, accompanied by thickening of the skin and subcutaneous nodules or masses that were firm and tender. There was a discharge containing black granules. An X-ray was performed and showed no abnormalities (Figure 1).

**Figure 2:**
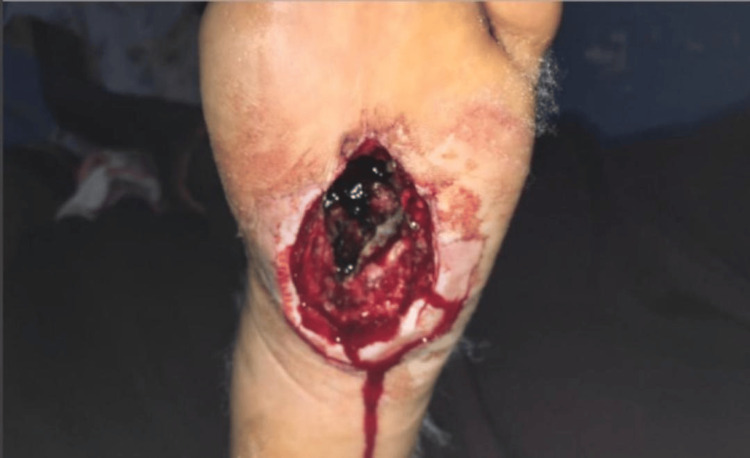
Aggressive debridement performed on the patient

**Figure 3:**
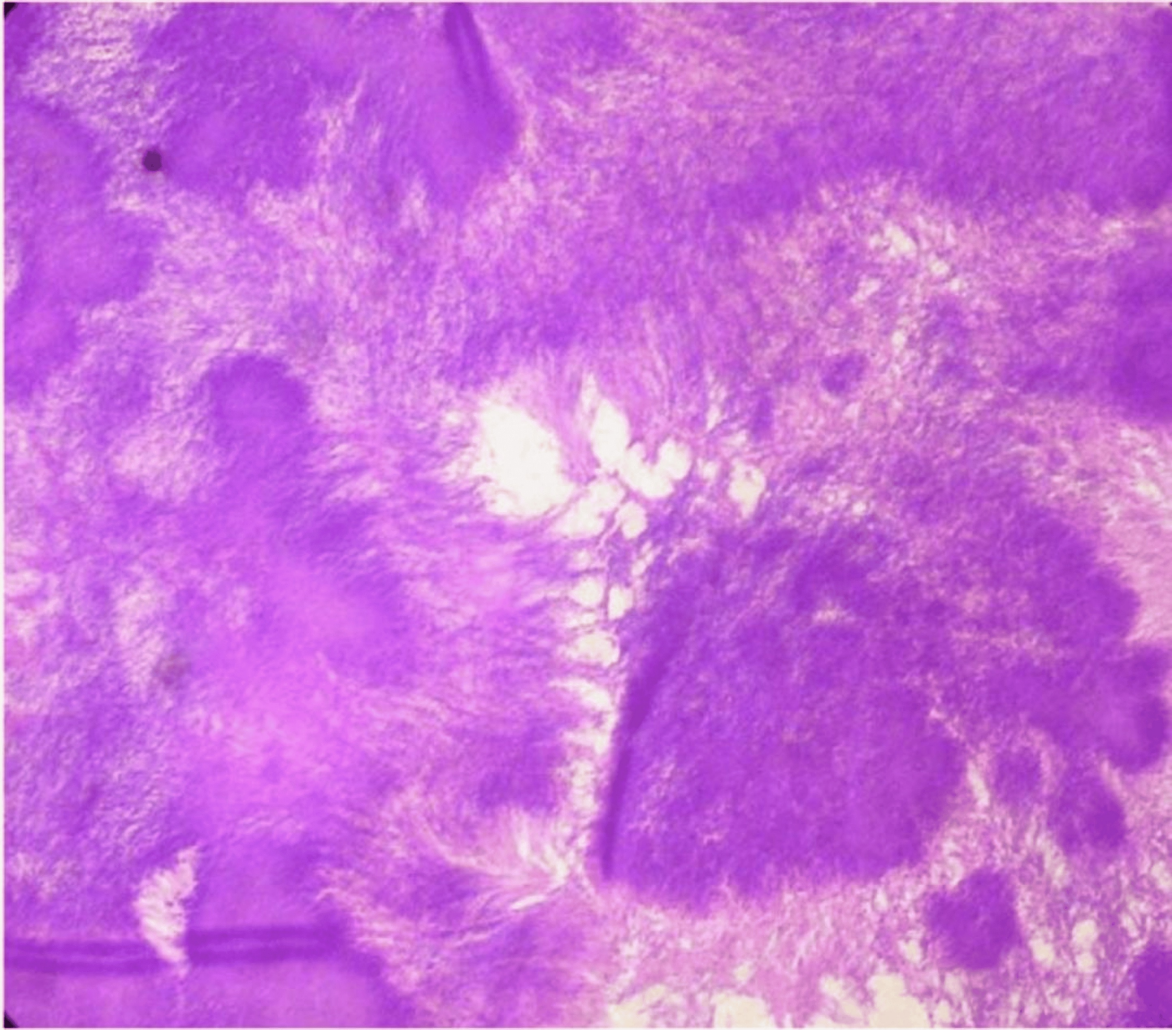
Granulomas with central fungal filaments (H&E, ×40)

**Figure 4:**
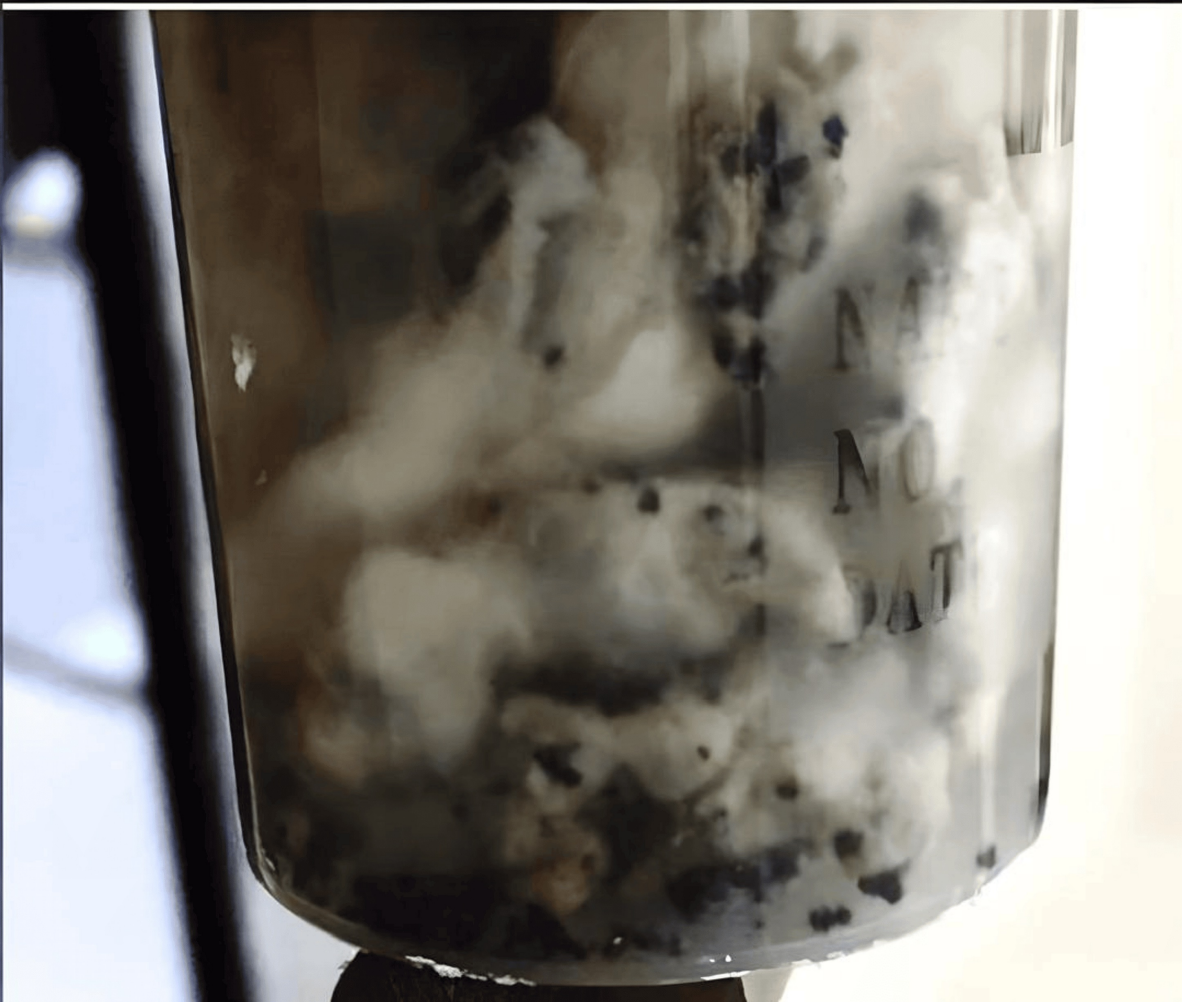
Patient’s tissue showing black granules of Madurella mycetomatis

Deep surgical debridement was performed involving the soft tissue at the plantar aspect of the foot, including the plantar fascia and plantar flexors of the toes (Figure 2). A fungal infection caused by Madurella mycetomatis was identified through histological examination of the tissue biopsy (Figure 3 and Figure 4).

**Figure 5:**
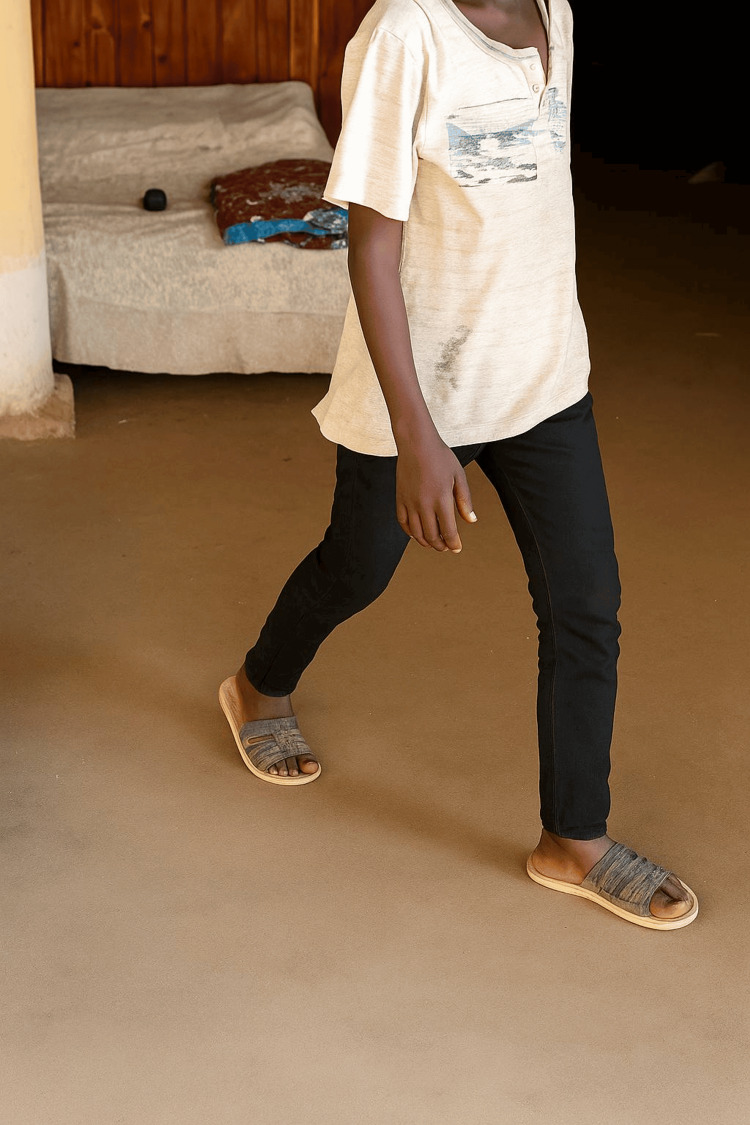
The patient regained normal function

After consistent wound care and administration of itraconazole at a dosage of 100 mg orally for antifungal treatment, combined with a broad-spectrum antibiotic, the patient achieved complete recovery, with no recurrence of sinuses or swelling. The patient resumed normal function (Figure 5) and was followed up for nine months, using ELISA, LFT, and RFT to monitor his condition.

Currently, the best diagnostic definition can be obtained through CT or MRI imaging, which provides differential diagnoses of the swelling and a more accurate assessment of the degree of bone and soft tissue involvement [3]. However, due to financial constraints, this was not performed.

The patient experienced two years of misdiagnosis and ineffective management, including two drainage procedures, without clinical improvement. Notably, the lesion remained non-tender throughout the course of the illness.

## Discussion

Mycetoma is a significant public health concern in tropical countries [4]. Healthcare professionals should consider this pathology in the differential diagnosis when patients present with persistent swellings, particularly in the foot, especially in tropical areas. The disease primarily affects individuals between 20 and 40 years of age, with a predominance of males and rural localization [5]. Madura foot, which accounts for approximately 70% of all mycetoma cases, is a disorder frequently contracted through a puncture wound or incision in the foot, especially in barefoot patients [6]. The arms, hands, and legs may also be affected [6].

Both bacteria and fungi can cause mycetoma. Microaerophilic actinomycetes can cause actinomycetoma, while true fungi can cause eumycetoma [7]. Histological examination of skin samples can identify the causal agent, which is essential for successful treatment [7]. A bacterial infection (actinomycetoma) is indicated by white, yellow, or red grains, whereas a fungal infection (eumycetoma) is indicated by black or white granules [7]. Additional imaging should be used to detect bone abnormalities after mycetoma is diagnosed [2]. Multiple non-specific osteolytic lesions, such as reactive sclerosis, cavities, and geodes, may be seen on X-ray imaging as signs of bone destruction [2]. Single or multiple thick-walled cavities with hyper-reflective echoes and no acoustic enhancement are typically observed on ultrasound and are frequently located toward the lower portion of the enlarged mass [2].

For the best characterization of the infection, differential diagnosis of edema, and precise evaluation of the extent of bone and soft tissue involvement, CT or MRI imaging is currently the reference standard [2].

Medical and surgical approaches must be used together to treat mycetoma [8]. Thorough surgical removal of affected tissue should be combined with antibiotic therapy (e.g., cotrimoxazole, amikacin, or minocycline) or antifungal therapy (e.g., ketoconazole or itraconazole) [8]. Disease recurrence is relatively common, occurring in around 50% of cases even with these treatments [9]. Post-surgical rehabilitation is crucial to restore functional mobility and improve quality of life, equipping patients who underwent amputation with a properly fitting prosthesis [9]. In tropical and subtropical regions, especially those with annual rainfall above 2500 mm, mycetoma is a prevalent infection [10].

## Conclusions

In tropical and subtropical climates, especially those with heavy annual rainfall above 2500 mm, mycetoma is a prevalent infection. This case report highlights that, if diagnosed and managed appropriately, patients can regain full function. Therefore, to prevent delayed diagnosis and the resulting functional and aesthetic complications, mycetoma should be considered in the differential diagnosis of chronically swollen feet.
